# Involvement of Epidermis Cell Proliferation in Defense Against *Beauveria bassiana* Infection

**DOI:** 10.3389/fimmu.2021.741797

**Published:** 2021-09-16

**Authors:** Wuren Huang, Ruijuan Tang, Shirong Li, Ying Zhang, Rongbing Chen, Liyuan Gong, Xuefei Wei, Yingyu Tang, Qiuning Liu, Lei Geng, Guoqing Pan, Brenda T. Beerntsen, Erjun Ling

**Affiliations:** ^1^Key Laboratory of Insect Developmental and Evolutionary Biology, CAS Center for Excellence in Molecular Plant Sciences, Shanghai Institute of Plant Physiology and Ecology, Chinese Academy of Sciences, Shanghai, China; ^2^Jiangsu Key Laboratory for Bioresources of Saline Soils, Jiangsu Synthetic Innovation Center for Coastal Bio-agriculture, Jiangsu Provincial Key Laboratory of Coastal Wetland Bioresources and Environmental Protection, School of Wetland, Yancheng Teachers University, Yancheng, China; ^3^The State Key Lab of Silkworm Genome Biology, Southwest University, Chongqing, China; ^4^Veterinary Pathobiology, University of Missouri, Columbia, MO, United States; ^5^Innovative Academy of Seed Design, Chinese Academy of Sciences, Beijing, China

**Keywords:** *Beauveria bassiana*, insect, cuticle, epidermis, infection, cell proliferation

## Abstract

Entomopathogenic fungi *Beauveria bassiana* can infect many species of insects and is used as a biological pesticide world-wide. Before reaching the hemocoel, *B. bassiana* has to penetrate the integument which is composed of a thick chitin layer and epidermal cells. Some chitinase, protease and lipase secreted by *B. bassiana* are probably involved in the fungal penetration of the integument. While microscopic proof is needed, it is difficult to locate the precise infection sites following the traditional method of immersion infection. Consequently, we developed a new method to inoculate conidia solution into a single fixed-site on the back of one segment. This fixed-site infection method is pathogenic but it is also dose dependent. Using the fixed-site infection protocol, it is also very convenient to track hyphae inside the cuticle layer by light and transmission electron microscopy. The fact that few hyphae were detected inside the chitin layer after fixed-site infection with mutant ΔBPS8, a protease secreted during fungi germination, indicates that this method is suitable for screening genes involved in penetrating the integument in large scale. We also found that melanization occurs before new hyphae penetrate the chitin layer. Most importantly, we discovered that fungal infection can induce epidermal cell proliferation through DNA duplication and cell division, which is essential for the host to defend against fungal infection. Taken together the fixed-site infection method may be helpful to determine the mechanism of fungal and host interaction in the integument so as to effectively exert fungal biological virulence.

## Introduction

*Beauveria bassiana*, an important entomopathogenic fungi, can infect most species of insects including economic insects like the silkworm and bee. Because it is safe to use and does not impact the environment or human lives, *B. bassiana* is widely used as a bio-pesticide (1, 2). Before reaching the hemocoel, *B. bassiana* must penetrate the integument, thereby initiating a successful infection. The insect cuticle can be successively divided into epicuticle, exocuticle, mesocuticle and endocuticle from the most outside to the inner side (3, 4). The cuticle is composed of chitin, lipid and proteins (5, 6). The protein and chitin interact with each other to form the hard and thick exoskeleton that prevents water evaporation and physical damage (7). On the epicuticle, the wax layer, which is mainly lipid and other carbohydrates, can prevent desiccation and provide chemical cues for species recognition (5, 6). As an entomopathogenic fungi, *B. bassiana* encodes many genes of chitinase, protease and lipase according to its genome (1, 2, 8). The production and secretion of some of those enzymes help to degrade the wax layer, chitin and proteins before *B. bassiana* can successfully penetrate the integument and invade the hemocoel (8).

There is very limited information available on fungus-host interactions in the integument as compared to what occurs in the hemocoel. *B. bassiana* conidia have a hydrophobic surface that can help to adhere to the insect cuticle through hydrophobic surface interaction (1, 2). In addition, the negative charge of the conidia and the composition of proteins and carbohydrates on the conidia surface can all affect the adherence of *B. bassiana* conidia to foreign surfaces (9). Subsequently, each conidia germinates on the insect epicuticle surface to form a germ tube with a appressorium at one end (1, 2). The appressorium further enlarges and forms the structure of a penetration peg. During this process, some genes have been identified that are involved in the initial infection. For example, two hydrophobin gene of *B. bassiana*, hyd1 and hyd2, are involved in conidia surface adhesion (2, 10, 11).

To date, studies of the interactions between entomopathogenic fungi and host are rather limited. It is known that fungi can secrete chitinase, protease and lipase to degrade and destroy the integument structure to permit penetration (1, 2, 8). On the other hand, the insect host may secrete some compounds into the integument to impact fungal infection (9). Additionally, it is unknown how various enzymes can cooperatively target the cuticle. In order to understand the key genes that are involved in the penetration of the integument, it is necessary to observe the invading hyphae of different mutants inside the cuticle using both light and transmission electron microscopy. In the laboratory, insects are usually suspended in a conidia solution for a short time to provide an opportunity for infection of the whole body (12, 13). However, because it is not possible to locate the exact infection sites where *B. bassiana* will penetrate the integument, it is very difficult to track the invading hyphae in the cuticle. As a substitute, grasshopper or locust wings or hydrophobic plastics have been utilized for conidia inoculation and the formation of appressoria and infection pegs (1, 2). However, insect wings and plastics are totally different from the integument and infections of these structures are likely very different than infections of the integument. Using *Frankliniella occidentalis* and *Verticillium lecanii* as a model, a group studied fungal penetration progress (14). The thickness of the *F. occidentalis* integument is approximately 0.5 μm, and the fungi can break through the integument very quickly and easily (14). The thickness of a typical lepidoptera larva is ~20 μm, which is quite a distance for *B. bassiana* to directly penetrate through. The mechanisms required for fungi to penetrate the thicker integument of a lepidoptera larva compared to the thinner integuments of *F. occidentalis* may be very different. Therefore, in order to understand the mechanism of insect integument penetration and the interaction of *B. bassiana* with its host on and inside the integument, it is necessary to develop a new infection method to track those invading hyphae at any time.

For the purpose of tracking *B. bassiana* we developed a fixed-site infection by spreading approximately 3 µl of conidia solution at a specific concentration on the top of the 5th integument (day1 of 5th instar) within an area of about 5 mm×5 mm. The results indicate that the fixed-site infection is pathogenic but it is dose-dependent, with conidia germination interrupted when the concentration of conidia was low. Following the fixed-site infection, it was convenient to track the invading hyphae in the cuticle by light and transmission electron microscopy. Deletion of BPS8, a fungal protease secreted during germination (15), weakens the penetration of cuticle, indicating that the fixed-site infection can be utilized to screen genes involved in fungi-host interactions on and inside the integument in large scale. Additional research indicated that fungal infection can induce epidermis cells to proliferate, which is essential for host defense against *B. bassiana* infection.

## Material and Methods

### Insects and Fungi

*Bombyx mori* (*Nistari*) was reared on mulberry leaves. Larvae on Day 1 of the 5th instar were used for all experiments in this study. The strain of fungal pathogen *Beauveria bassiana* ARSEF2860 was routinely maintained for the preparation of conidia (12, 16).

### Fixed-Site Infection

**Figure 1:**
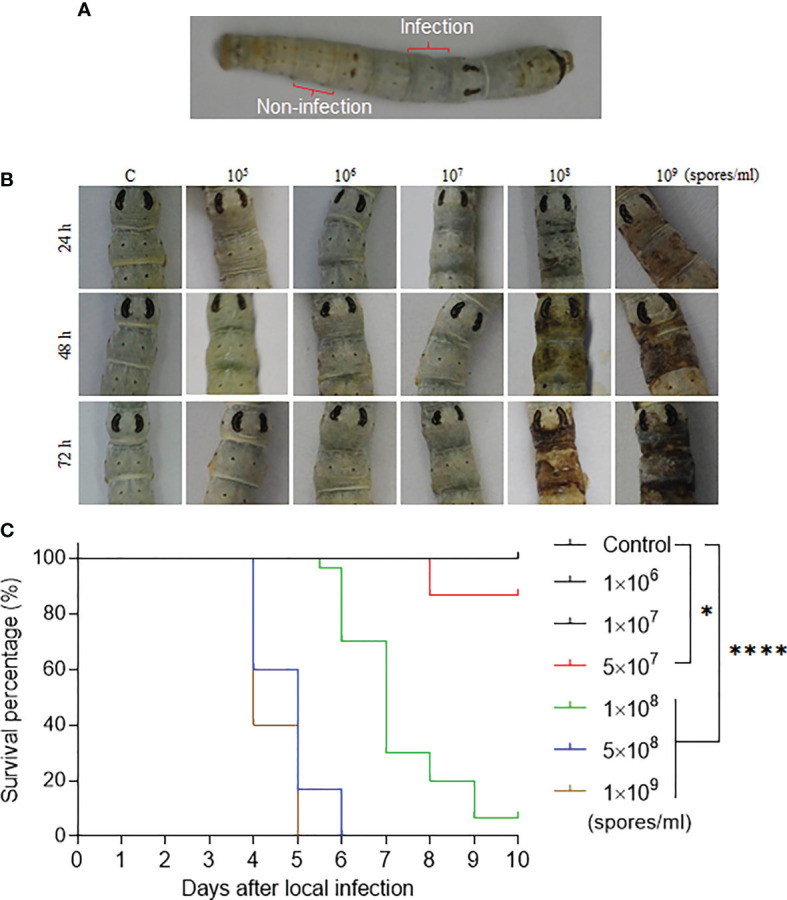
The fixed-site infection and its pathogenicity. **(A)** Development of the fixed-site infection. The washed and dried silkworm larvae on day 1 of the 5th instar stage were used in this study. The back of the 5th segment labeled as “Infection” is the area (approximately 5 mm× 5 mm) for inoculation with 3 µl of conidia solution at different concentrations. The conidia solution was spread evenly. The 8th segment, which is two segments away from the infection site, is labeled as “Non-infection” that will be sampled as a control if necessary. **(B)** Morphological changes of integuments after receiving the fixed-site infection. Conidia solutions at different concentrations were inoculated as described above. Morphological changes in the infection sites were observed and imaged at each indicated time point. **(C)** Bioassay after the fixed-site infection. Conidia solution (3 µl) at a concentration of 1×10^6^-1×10^9^ spores/ml was applied for fixed-site infection individually. Conidia solution at a concentration of 1×10^8^-1×10^9^ spores/ml was determined to be lethal to the larvae using the fixed-site infection model. Please note that conidia at 1×10^8^ spores/ml is usually used in a traditional infection route by submerging all larvae in a conidia solution for a period of time. Conidia at a concentration under 1×10^7^ spores/ml was not pathogenic. Kaplan-Meier survival curves were compared using the log-rank (Mantel-Cox) test among each concentration of infection and control (*p < 0.05; ****p < 0.0001).

Conidia were suspended in 0.05% Tween 20 (V/V) at different concentrations. The silkworm larvae were washed and dried using a paper towel and then 3 µl of conidia solution was dropped on the back of the 5th integument segment labeled as “Infection” in **Figure 1A**. Next a pipette tip was used to spread conidia solution over an area of approximately 5 mm×5 mm. The applied conidia solution was dried using an electric fan. The larvae that received the fixed-site infection were placed inside a separate room and maintained in a large box with 70-80% humidity. All larvae were fed mulberry leaves at 25°C with fresh leaves replaced as needed. The integuments labeled as “Infection” and “Non-infection”, as shown in **Figure 1A**, were sampled at different time points and corresponding integuments of naïve larvae were also sampled as a control. These samples were fixed as needed for tissue section or scanning electron microscopy or transmission electron microscopy.

### DNA Duplication and Cell Division

After the fixed-site infection, silkworm larvae, including naïve ones, were injected with 5-Bromo-2′-deoxyuridine (BrdU, Sigma, 19-160) at 10 µl/g body weight to label DNA duplication in epidermis cells for three hours (17). The primary antibody against BrdU (Sigma, B8434; 1:1000) was utilized to label cells that had incorporated BrdU as described in detail (17, 18). The antibody against Phospho-Histone H3 (PH3, Merck, H0412; 1:1000) was used to detect cells that were dividing (17). The secondary antibody conjugated with Alexa Fluor-488 or Alexa Fluor-594 was utilized and DAPI was used to counter-stain nuclei. Images were acquired using a fluorescence microscope with the corresponding filter (Olympus BX51, Japan).

### Scanning Electron Microscopy and Transmission Electron Microscopy

The methods of scanning electron microscopy and transmission electron microscopy were used as previously described (19) with some modifications. Briefly, for the scanning electron microscopy, the integuments were fixed in paraformaldehyde 4% for 48 h at 4°C followed by dehydration in different concentrations of acetone by turn (70, 80, 90, 95 and twice in 100%, for 10 min each). Next the samples were mounted in steel stubs with double-sided adhesive tape to be coated with gold in sputtering. The integuments were photographed using a scanning electron microscope Hitachi TM3000 (Hitachi Hight-Technologies Corporation/Japan). For the transmission electron microscopy, integuments were fixed with 2.5% glutaraldehyde and 1% osmium tetroxyde and embedded in Epon resin. Ultra-thin sections were stained with uranyl acetate and lead citrate and a JEOL 100CX transmission electron microscope was used for observation.

### Cisplatin Oral Feeding and Injection

In order to determine a proper Cisplatin concentration for these studies, this compound was co-cultured with *B. bassiana* conidia in SDB medium at different concentrations to determine a safe concentration that did not affect conidia germination and growth *in vitro*. In addition, Cisplatin at different concentrations was spread on mulberry leaves for feeding larvae that received the fixed-site infection or not to determine the Cisplatin concentration that did not affect growth of the naive larva or the occurrence of melanization upon infection. Ultimately 6.25 µg/ml of Cisplatin was selected as the safe concentration for feeding to the silkworm larvae. 10 µl of 6.25 µg/ml of Cisplatin was spread onto mulberry leaves (1 cm×1 cm), which were food for the silkworm larvae that received the fixed-site infection or not. Fresh leaves containing Cisplatin were replaced when necessary. The same size of mulberry leaves spread with buffer were fed as a control.

After entering the 5th larval stage, no DNA duplication and cell division were detected in epidermis cells (17). DNA duplicates in the midgut cells during 4th larval stage. In order to understand whether Cisplatin can inhibit DNA duplication, larvae on Day 1 of the 4th instar were injected with 1, 3 and 7 μg of Cisplatin including buffer as the control. BrdU was injected as described above three hours before the midguts were dissected. DNA duplication was detected as above.

### Hemolymph Sample and Prophenoloxidase Activity Assay

Different concentrations of Cisplatin were spread on pieces of mulberry leaves and fed to the silkworm larvae as above. At the scheduled time, hemolymph of each single larva was collected on ice separately. Next all hemolymph was centrifuged at a speed of 8000 g at 4°C for 5 min. The supernatant was collected separately for assaying plasma phenoloxidase (PO) activity with some modification (20). Briefly 5 μl of plasma was added to 400 μl L-DOPA (2 mM) dissolved in 10 mM Tris buffer (pH 7.5). The absorbance was continuously read at 490 nm using the EXPERT 96 microplate reader (Biochrom, Holliston, MA, USA). One unit of PO activity was defined as ΔA_490_/min=0.001.

### *In Situ* Apoptosis Detection: The TUNEL Method

The One Step TUNEL Apoptosis Assay Kit (Beyotime, China) was used to check for apoptotic cells in epidermis that had received a fixed-site infection or not following the manufacturer’s instructions. DAPI was used to counter-stain nuclei for fluorescent microscopy.

### Statistical Analysis

Survival data were plotted using the Kaplan–Meier method and comparisons among groups were made using the log-rank (GraphPad Prism). In all tests, P ≤0.05 was considered significant.

## Results

### Pathogenicity of *B. Bassiana* Infection on the Fixed Site

**Figure 2:**
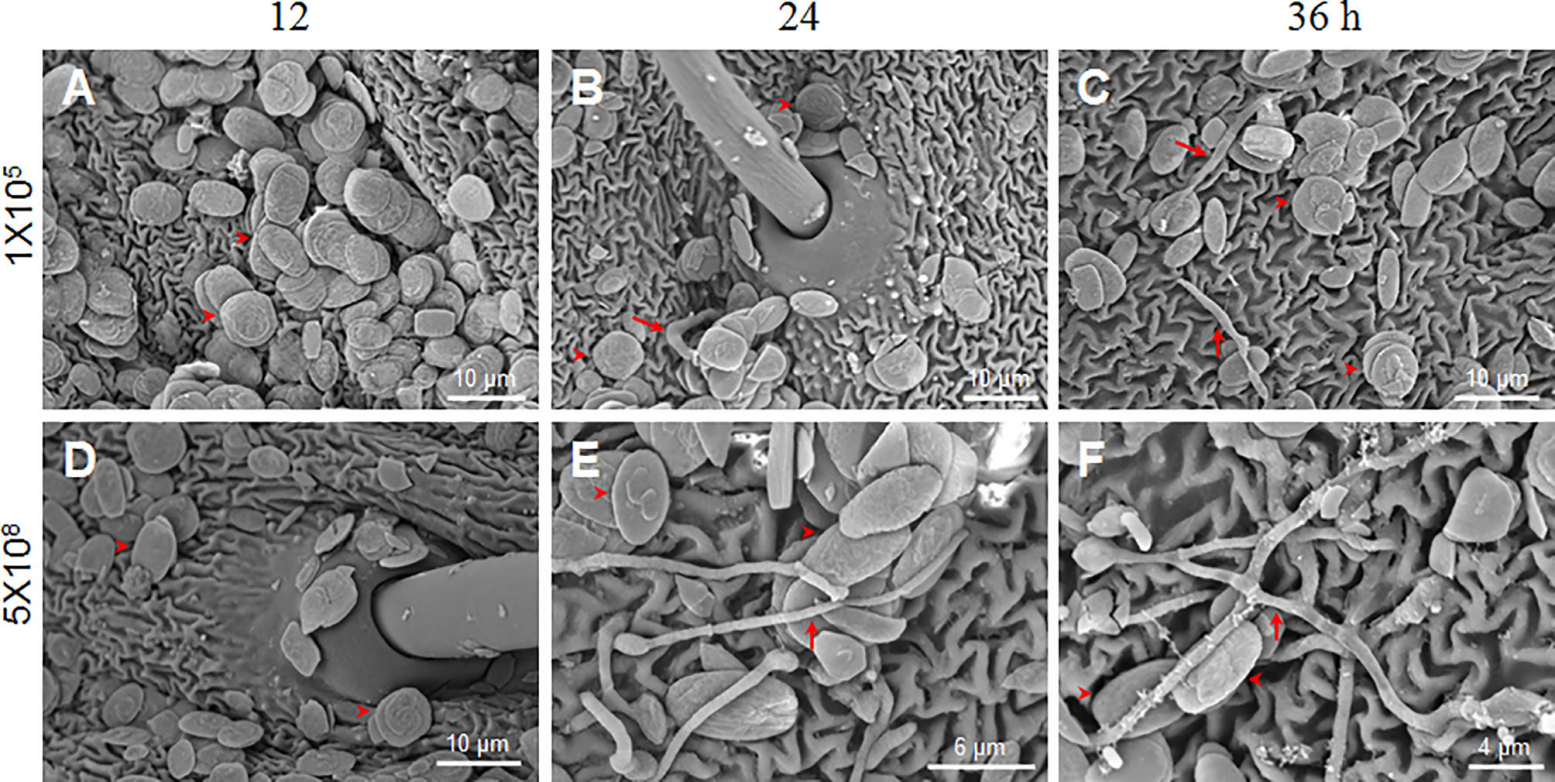
*B. bassiana* conidia germination is dose-dependent. Scanning electron microscopy was utilized to observe conidia germination after the fixed-site infection (1×10^5^ and 5×10^8^ spores/ml) at each indicated time point. In general, the number of conidia detected with 1×10^5^ spores/ml **(A–C)** was less than 5×10^8^ spores/ml **(D–F)** using scanning electron microscopy. To obtain additional information, we specifically selected the area with many conidia for imaging when 1×10^5^ spores/ml of conidia was inoculated. Few conidia germinated and mycelia were thin as indicated by the arrows **(B, C)**. When 5×10^8^ spores/ml of conidia were inoculated, many conidia germinated and new hyphae were thick on the integument surface **(E, F)**. At 12 h, no germination occurred when either concentration of conidia was inoculated **(A, D)**. In each picture, the arrowheads indicate some conidia likely without germination.

*B. bassiana* is an important entomopathogenic fungi that can infect many species of insects at the larval and/or adult stages. In the laboratory, *B. bassiana* is an important model to study the interaction of fungi and host following topical infection *via* suspension of insects in the conidia solution (12, 16). So, using this method, the infection sites are random and cannot be exactly predicted until melanized spots are left on the surface where *B. bassiana* penetrated the integument and reached the hemocoel. In order to understand the process of penetration, we developed a new infection method by spreading 3 µl of spore suspension on the top of the 5th segment (approximately 5 mm×5 mm) as indicated in **Figure 1A**. We call it the fixed-site infection in this study thereafter. The eighth segment, which is separated by two segments from the fixed-site (5th segment), was sampled as a control. We first performed a dose response for infection (1×10^5^ to 1×10^9^ spores/ml) that was observed and imaged at different time points. The results indicate that 1×10^8^-1×10^9^ spores/ml could induce melanization on the fixed-site after infection but the time to appearance was different (**Figure 2B**). The higher conidia concentrations used, the earlier melanization appeared. Bioassays demonstrated that 1×10^8^-1×10^9^ spores/ml of conidia used for the fixed-site infection are pathogenic (**Figure 1C**). Conidiae at 5×10^7^ spores/ml or higher concentration could significantly induce infection. However, a concentration of approximately 1×10^6^-1×10^7^ spores/ml did not induce infection at all. In many papers, a concentration of around 1×10^7^-1×10^8^ spores/ml are usually used for topical infection. Therefore, the pathogenicity of 1×10^8^ spores/ml by either traditional suspension or fixed-site infection is comparable. These results demonstrate that the fixed-site infection by *B. bassiana* is pathogenic but it also depends on the concentration of conidiae used.

### Spore Germination is Dose-Dependent

*B. bassiana* at a concentration of 1×10^7^ spores/ml had no obvious pathogenicity after fixed-site infection (**Figure 1**). We were curious why such a high conidia concentration could not induce infection in the larvae. Therefore, we inoculated conidiae at 1×10^5^ and 5×10^8^ spores/ml using the fixed-site infection model and observed the morphological changes of those conidiae at different time points using scanning electron microscopy. The results demonstrate that no germination occurred at 12 h after spore inoculation (**Figures 2A, D**). When conidiae at 1×10^5^ spores/ml were inoculated, very few mycelia were detected on the surface at 24 h (**Figure 2B**) and 36 h (**Figure 2C**), respectively. In addition, those mycelia appeared to be dehydrated. On the contrary, when a higher concentration at 5×10^8^ spores/ml was inoculated, many conidiae germinated and thick mycelia were detected at 24 h (**Figure 2E**) and 36 h (**Figure 2F**). At this concentration, there were still conidiae that were not germinated between 24-36 h. The results demonstrate that *B. bassiana* conidiae germination is dose-dependent. Very few conidiae could germinate if a low concentration of conidiae was applied, which can explain the reason that pathogenicity is limited if low concentrations of conidiae are applied for the fixed-site infection.

### Tracking of *B. Bassiana* Hyphae Invasion in the Integument After Fixed-Site Infection

**Figure 3:**
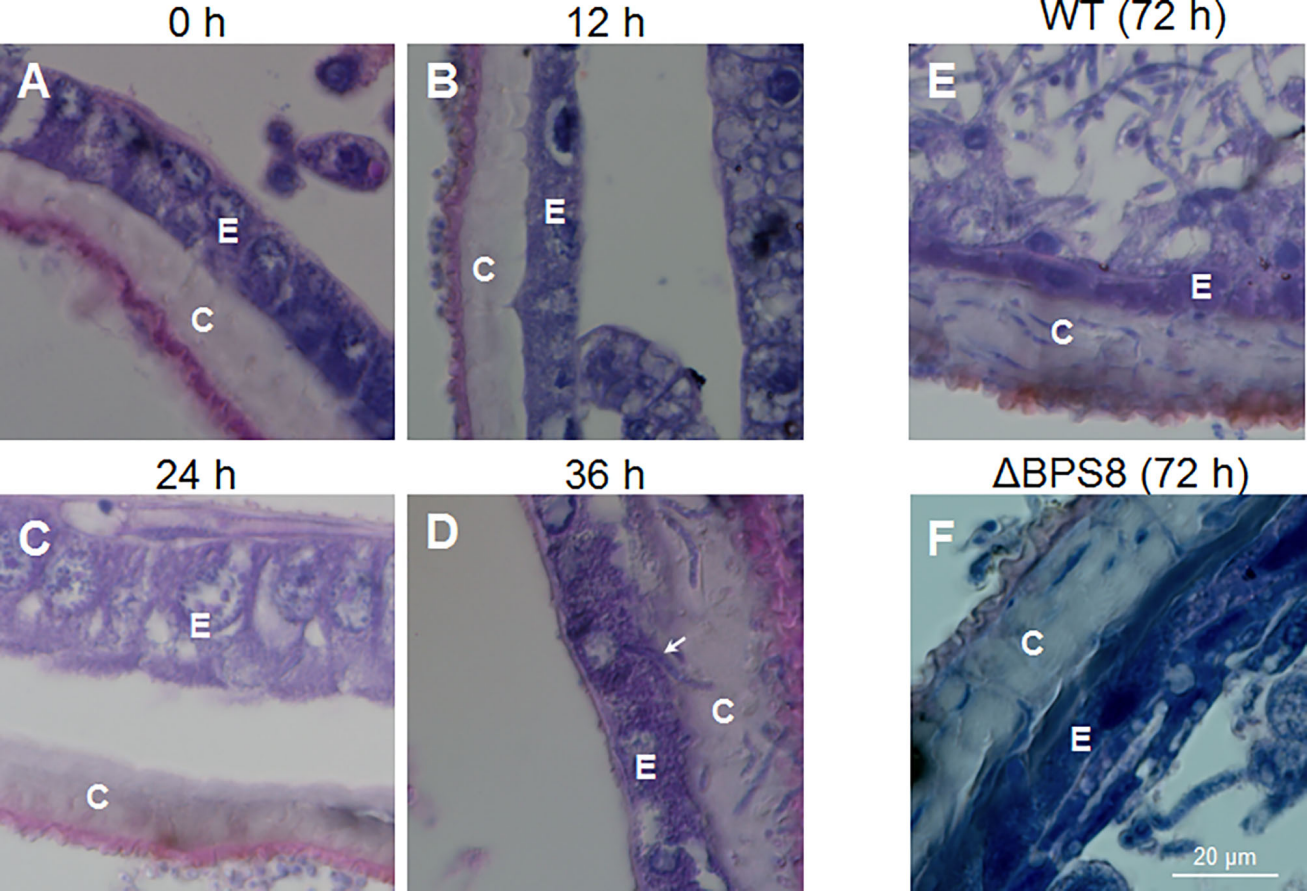
*B. bassiana* hyphae inside the cuticle using light microscopy. After initiating the fixed-site infection (1×10^8^ spores/ml, the same with the following work unless otherwise mentioned), the integuments were sampled *via* tissue section and hematoxylin-eosin staining at different time points. BPS8 is a protease secreted by *B. bassiana* during germination (15) and conidia of the ΔBPS8 mutant (with BPS8 deleted) were also inoculated as above and sampled at 72 h. No hyphae were detected inside the cuticle within the first 24 h **(A–C)**. Melanized cuticle was detected at 24 h **(C)**. At 36 h **(D)**, many hyphae inside the cuticle were clear and the arrow points to a hypha that was ready to penetrate the nucleus of an epidermal cell **(D)**. At 72 h **(E)**, the number of hyphae inside the cuticle increased and hyphae were detected in the hemocoel. At 72 h, only a few hyphae of ΔBPS8 were detected inside the cuticle **(F)**, which was fewer than wild type *B*. *bassiana*, and additionally few hyphae of ΔBPS8 were detected inside the hemocoel. In each picture, the small letter “E” and “C” represent epidermis cells and cuticle respectively.

It is difficult to locate exact infection sites using the traditional infection method of suspension unless melanization occurred to indicate penetration sites. We suspected, however, that it may be much easier to observe invading hyphae in the integument after the fixed-site infection method using light and transmission electron microscopy. To confirm this suspicion, a concentration of 1×10^8^ spores/ml was applied for the fixed-site infection. The infected segments were sampled at different time points for tissue section and hematoxylin-eosin staining. The results show that there were no penetrating hyphae prior to 0-24 h after the fixed-site infection (**Figures 3A–C**). At 24 h, some cuticles were melanized (**Figure 3C**), which matches the morphological changes previously observed (**Figure 1B**). At 36 h, many hyphae were observed inside the cuticle (**Figure 3D**). A hypha was even ready to penetrate the nucleus of an epidermis cell (arrow-indicated in **Figure 3D**), indicating that it is possible for *B. bassiana* to cause physical damage to the epidermal cells during infection. At 36 h, few hyphae were detected in the hemolymph. At 72 h, there were many more hyphae detected in the cuticle (**Figure 3E**). In the meanwhile, free hyphae appeared in the hemocoel. Entomopathogenic fungi can secrete chitinase, protease and lipase to degrade the insect cuticle structure during infection (1, 2, 8). Previously we demonstrated that *B. bassiana* conidiae secrete BPS8 during germination (15). And the insect hosts can hijack BPS8 to activate prophenoloxidase (PPO) remaining in the dried molting fluids to inhibit conidiae germination (15). BPS8 is a toxic factor since ΔBPS8 mutant killed silkworm larvae in a delayed way when compared with the wild type (15). The same concentration of ΔBPS8 mutant conidiae was applied for the fixed-site infection. At 72 h, a very limited number of hyphae were detected in the cuticle and hemocoel (**Figure 3F**) when compared with the wild type (**Figure 3E**). This result indicates that BPS8 has a close association with integument penetration when the fixed-site infection assay is used.

**Figure 4:**
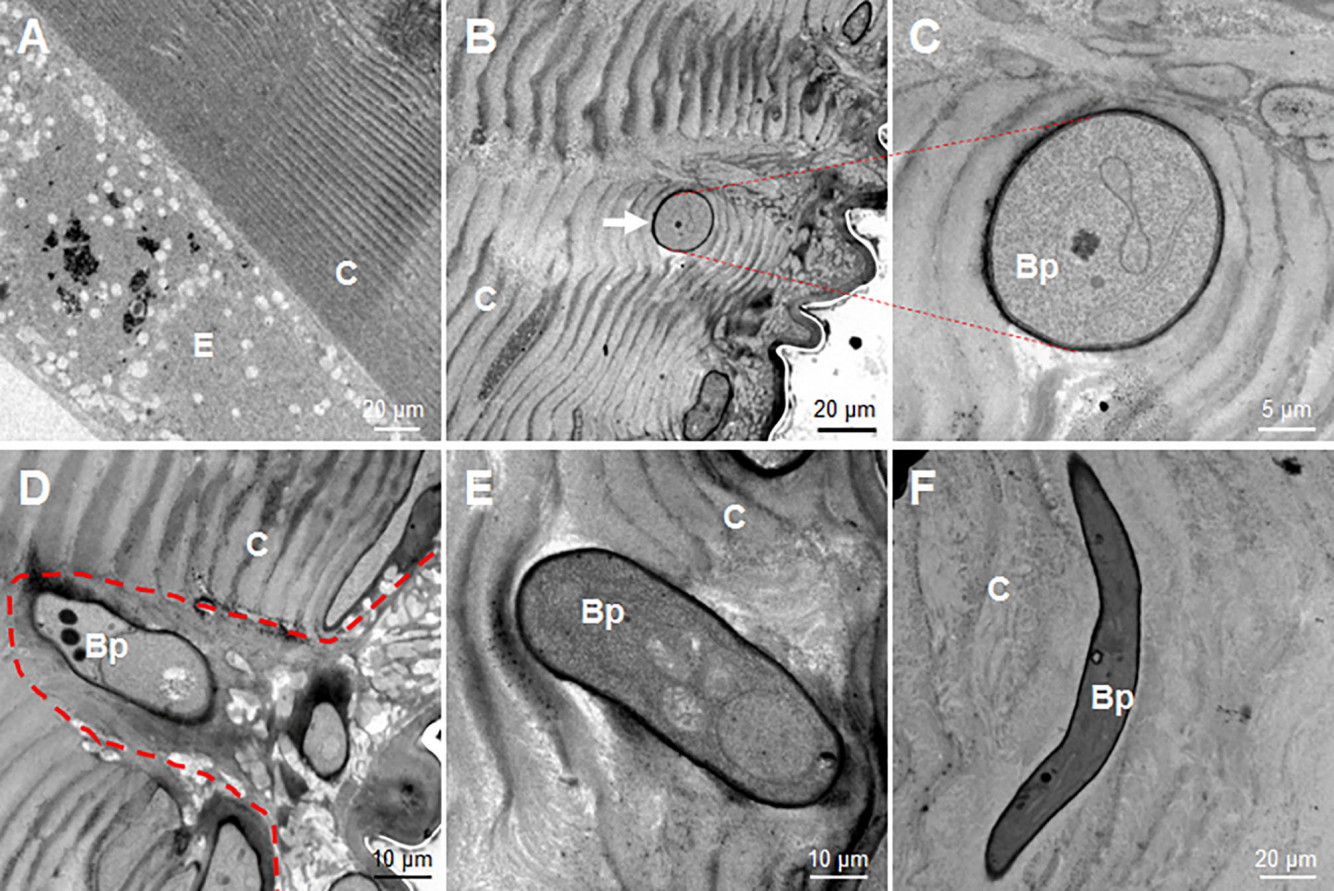
Morphology of *B. bassiana* hyphae using transmission electron microscopy. After the fixed-site infection, the infected integuments and the naive ones were sampled at 36 h for observation *via* transmission electron microscopy. **(A)** Morphology of cuticle and epidermis cells from a naive larva. The structure of the cuticle was regular in the naive cuticle. **(B–F)** Hyphae in the cuticle of larvae that received the fixed-site infection. **(B, C)** A hyphae penetrated its way along two pieces of cuticle lamina and was sectioned vertically. The enlarged intersecting surface of this hypha and its surrounding cuticle lamina are shown in **(C)**. **(D)** A newly-developed hypha degraded the cuticle to form an empty cavity at the beginning of infection. **(E, F)** Two hyphae inside the cuticle far from the epicuticle. They penetrated in different directions without further degradation of the surrounding cuticle. In each image, the small letter “E” and “C” represent epidermis cells and cuticle respectively.

At 36 h, the fixed-site infected and non-infected segments were sampled respectively for observation *via* transmission election microscopy. In the control naive larvae, the laminae of the cuticle were regularly distributed, and the epidermal cells were also in good condition (**Figure 4A**). However, after the fixed-site infection, the invading hyphae were observed in the cuticle to penetrate in different directions (**Figures 4B–F**). For example, a hypha was observed to push its way between cuticle laminae in a direction vertical to the tissue section (**Figure 4B**). An enlarged image shows two pieces of cuticle laminae that were pushed aside and became curved (**Figure 4C**). During the initiation of infection, the new hypha degraded the neighboring epicuticle extensively (**Figure 4D**). After entering the cuticle, the hyphae may depend on physical pressure derived from growth to penetrate in different directions (**Figures 4E, F**). These results demonstrate that the fixed-site infection is convenient for observing the invading hyphae inside the cuticle. In addition, the fixed-site infection model may be feasible for screening genes that are directly or indirectly involved in integument penetration and subsequent growth inside as demonstrated by the ΔBPS8 results that showed weak penetration of the integument (**Figure 3F**).

### Fungal Infection Induces Epidermis Cell Proliferation

**Figure 5:**
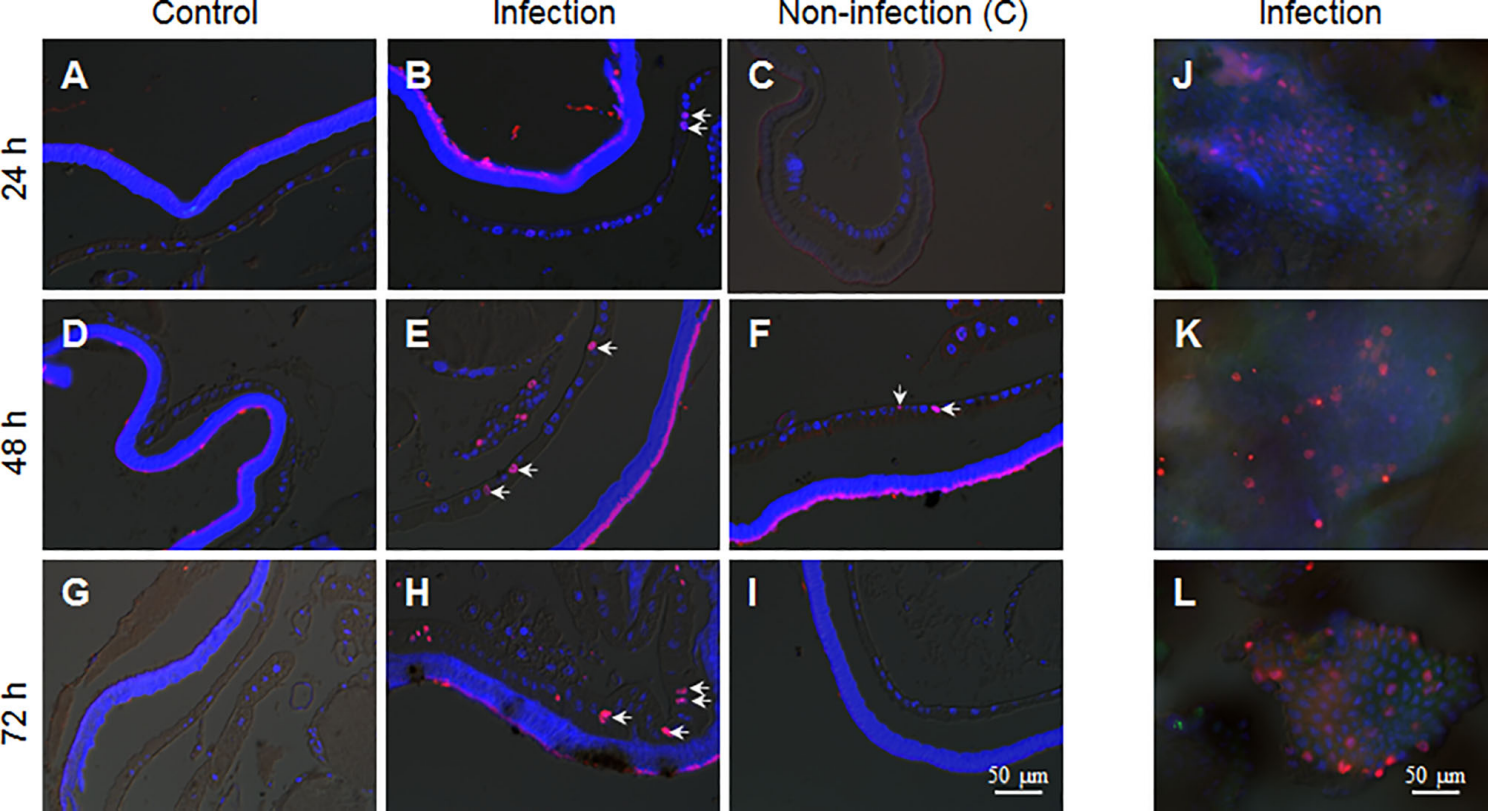
*B. bassiana* infection induces DNA duplication in epidermis cells. BrdU was injected 3 h before sampling. The integuments that received the fixed-site infection were sampled *via* tissue section or immuno-staining directly. The non-infection integuments, as indicated in **Figure 1A**, were also sampled. **(A, D, G)** No DNA duplication was detected in epidermis cells of naive larvae. **(B, E, H)** The fixed-site infection induced DNA duplication in epidermal cells at each time point assayed. Some of the BrdU-positive nuclei (Red) are indicated by arrows. Some epidermal cells of non-infection segments incorporated BrdU especially at 48 h after infection **(C, F, I)**. **(J–L)** Direct staining shows the number of BrdU-positive cells in the integuments that received the fixed-site infection. Nuclei were counter-stained by DAPI.

Epidermis cells of larvae in the 5th larval stage have no DNA duplication and cell division (17). Because during integument penetration, the invading hyphae may physically damage some epidermal cells (**Figure 3D**), we wanted to know whether the fixed-site infection can induce cell proliferation in epidermis cells. After performing the fixed-site infection as described above, the segments were either infected (**Figures 5C, F, I**) or not-infected (**Figures 5B, E, G**) as indicated in **Figure 1A**, were sampled at different time points (24, 48, and 72 h) with BrdU injected 3 h prior to sampling to demonstrate DNA duplication. The corresponding segments of naive larvae were sampled as a control (**Figures 5A, D, G**). The results show that some epidermal cells demonstrated DNA duplication at 48-72 h according to BrdU-positive staining (**Figures 5B, E, H**). BrdU-positive cells were also detected in non-infection integuments at 48 h (**Figure 5F**), indicating that the BrdU-incorporation signal that was induced by fungal fixed-site infection is far-reaching. No BrdU-incorporation was detected in the epidermis cells of control segments (**Figures 5A, D, G**). To confirm the above results, each piece of infected integument was directly applied for BrdU-staining. These results show that there were many epidermis cells that had incorporated BrdU since 24 h after the fixed-site infection (**Figures 5J–L**). Compared with wild type, the penetration of the epidermis by the ΔBPS8 mutant was less after the fixed-site infection (**Figure 3F**). Correspondently, DNA duplication induced by the ΔBPS8 mutant was also less (**Figure S1**). Taken collectively, all of the above results show that the fixed-site infection by *B. bassiana* can induce DNA duplication in epidermis cells, which is an important part of cell proliferation.

**Figure 6:**
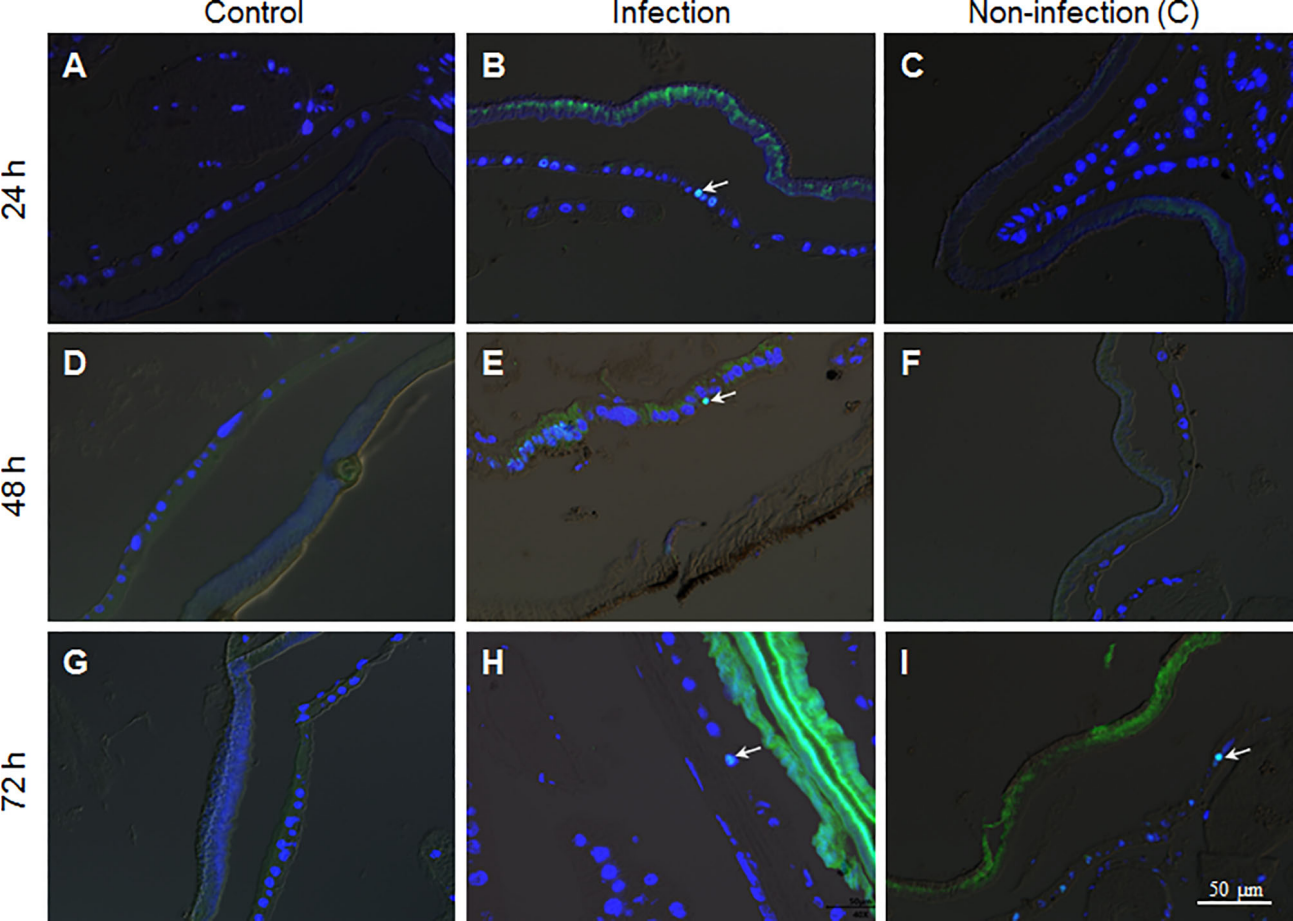
The fixed-site infection by *B. bassiana* promotes division in some epidermis cells. Samples were prepared as shown in **Figure 5** and antibodies against the cell division marker PH3 were applied for immuno-staining. **(A, D, G)** No epidermal cell division was detected in the naive integument. **(B, E, H)** After the fixed-site infection, some epidermis cells were PH3-positive (Green and arrow-point) at each time point assayed, indicating those epidermis cells undergoing division. **(C, F, I)** In the non-infection integuments, PH3-positive cells were also detected, particularly at 72 h after infection (arrow-point). Nuclei were counter-stained by DAPI.

To confirm if there were epidermal cells undergoing division, PH3 antibody, the mitosis marker, was applied during immuno-staining of the fixed site integument and the non-infection integument. The results show that there were no dividing epidermis cells from naive larvae at any time assayed (**Figures 6A, D, G**). However, a few epidermis cells were undergoing division if the integuments received the fixed-site infection (**Figures 6B, E, H**). Epidermis cell division was observed in the non-infection integuments at 72 h (**Figure 6I**) but not at 24 and 48 h (**Figures 6C, F**). Just like DNA duplication, the signal to induce epidermis cell division is also far-reaching after the fixed-site infection. After fixed-site infection, apoptotic cells were not detected in the epidermis (**Figure S2**). Additionally, no necrosis in epidermis was detected after direct staining using Propidium iodide. Therefore, epidermis cell proliferation is not due to apoptotic cells caused by fixed-site infection. These data demonstrate that *B. bassiana* surface infection can induce epidermis cells to proliferate by unknown factors with the influence transferred to integument segments far away from the infected sites.

### Epidermis Cell Proliferation is Involved in Defending Against *B. Bassiana* Infection

**Figure 7:**
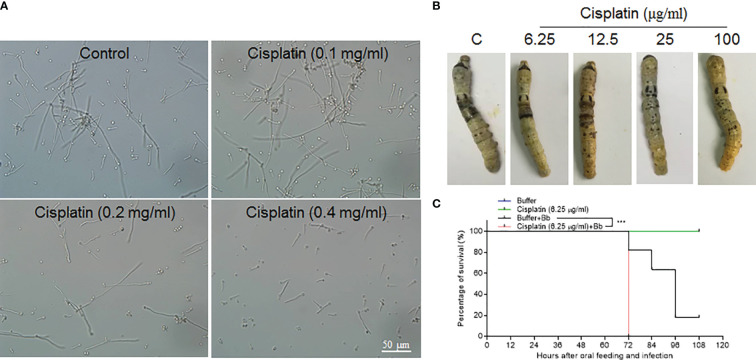
Epidermis cell proliferation is an important host response against *B. bassiana* infection. Due to lacking effective genetic manipulation to inhibit epidermis cell proliferation, Cisplatin, the inhibitor of cell DNA duplication and division (21, 22), was fed to larvae that received the fixed-site infection or not. **(A)** Screening to determine a proper concentration of Cisplatin that did not affect *B. bassiana* conidia germination and growth. Cisplatin <0.1 mg/ml did not impact *B. bassiana* growth. **(B)** Screening to determine a suitable concentration of Cisplatin that does not affect melanization after the fixed-site infection for 48 h. Ultimately, it was determined that 6.25 µg/ml of Cisplatin did not interrupt *B. bassiana* (indicated as Bb in the graph) development and integument melanization and this amount was spread on mulberry leaves to feed to larvae after the fixed-site infection. **(C)** Bioassay after the fixed-site infection with or without Cisplatin fed to inhibit epidermis cell proliferation. Cisplatin (6.25 µg/ml) fed to larvae without fungal infection did not induce any death. After the fixed-site infection, Cisplatin feeding significantly increased *B. bassiana* virulence with most larvae dying at 72 h after infection. Without Cisplatin feeding, infected larvae lived a significantly longer time. Kaplan-Meier survival curves were compared using the log-rank (Mantel-Cox) test after the fixed infection among with Cisplatin involved or not (***p = 0.002).

In lepidoptera, there is still no convenient and effective genetic method to specifically manipulate epidermis cell division alone without affecting other tissues. Cisplatin may interrupt cell DNA duplication and cell division (21, 22). To confirm that Cisplatin can inhibit DNA duplication in the silkworm, small amounts of Cisplatin were injected into larvae at the 4th larval stage when DNA duplication occurs in large scale in midgut cells The results indicate that Cisplatin inhibits DNA duplication within a short time period in a dose dependent manner (**Figure S3**). Therefore, Cisplatin is likely suitable for inhibiting cell proliferation during fungal infection. Before oral feeding the silkworm larvae with Cisplatin, *B. bassiana* conidiae were co-cultured with Cisplatin at different concentrations to determine the appropriate dose without or with limited influence on *B. bassiana* development. The results indicate that Cisplatin less than 0.1 mg/ml did not influence *B. bassiana* growth (**Figure 7A**). Cisplatin (< 0.1 mg/ml) was spread on mulberry leaves for feeding the naive silkworm larvae and no influence on the larval growth was observed (data not shown). In order to determine the proper concentration for use, different concentrations of Cisplatin were fed to the silkworm larvae that received the fixed-site infection (**Figure 7B**). After the fixed-site infection for 48 h, epidermal melanization was inhibited if the concentrations of Cisplatin fed were increased. We then decided to check whether Cisplatin feeding can affect plasma PO activities. Without fungal infection, Cisplatin feeding at different concentrations did not significantly inhibit plasma phenoloxidase (PO) activities if compared with the control (**Figure S4**). Furthermore, plasma PO activities among different Cisplatin feeding were almost at the same level, indicating that the inhibition on the infected sites melanization in **Figure 7B** was not due to Cisplatin feeding. It is known that melanization can enhance the reactive oxygen species (ROS) level. Consequently, in order to determine if ROS directly induce epidermis cells to proliferate, N-Acetyl-L-cysteine (NAC) and vitamin C, which are usually utilized to decrease the ROS level (23, 24), were fed to the larvae that received the fixed-site infection. Following this treatment, no significant change in epidermis cell proliferation was observed, indicating that melanization does not have an impact. Eventually Cisplatin at 6.25 µg/ml was selected for spreading on mulberry leaves, which then were fed to the silkworm larvae that either received or not the fixed-site infection. The results indicate that Cisplatin feeding induced the silkworm larvae to die significantly quicker than those without Cisplatin feeding (**Figure 7C**). Most larvae that received the fixed-site infection with Cisplatin feeding died at 72 h. These results indicate that epidermis cell proliferation is very important for hosts to defend against fungal infection.

## Discussion

In the laboratory, insects are usually suspended in *B. bassiana* conidia solution for a whole-body topical infection (12, 16). According to scanning electron microscopy, conidiae of *B. bassiana* and *Metarhizium anisopliae* are usually found to accumulate in the spiracle, hair sockets, pores of the wax gland, sensory bits and articulating membrane of the legs (19). Entomopathogenic fungi were detected germinating in those areas and penetration of the integument occurred soon afterwards. Our fixed-site infection is very similar to accumulation of conidia in or around those tissues. The integument we selected on the back of the 5th segment (on day 1 of the 5th instar) has a thick cuticle layer that may not be easy for *B. bassiana* to penetrate. However, this feature allows a long time window to observe *B. bassiana* penetration of the integument. Ultimately, we showed that the fixed-site infection is as feasible to induce pathogenicity as is the traditional infection method by submerging larvae in the conidia solution.

Utilizing this newly-developed method, some unexpected results were obtained that likely would have been rarely observed if the traditional method were utilized for infection. It is expected that most conidiae attached to the insect surface may germinate if the proper moisture level is maintained. However, very few conidia germinated if 1×10^5^ spores/ml were inoculated and permitted to infect (**Figures 2A–C**). Therefore, not all conidia can germinate and take part in an infection. Currently, the exact mechanism of dose-dependent germination is unknown but it is under investigation. The typical phenomenon after fungi infection is the appearance of melanized spots on the integument at three days following infection (12, 16). However, our results indicate that melanization already had occurred before hyphae developed and penetrated the cuticle (**Figure 3C**). In the case of traditional infection, the infection sites may not be large enough and the melanized spots are likely very small and invisible in the beginning. With the fixed-site infection model, the melanized spots are enlarged and become visible immediately following occurrence.

It is believable that fungi secrete many enzymes to degrade the topical wax and chitin during the infection process. The transmission electron microscopy observations indicate that there was cuticle degradation at the beginning of penetration (**Figure 4D**). After entering the cuticle, the new hyphae appear not to degrade the around cuticle further as did near epicuticle (**Figures 4B, E, F**). At this stage, the hyphae are likely to push their way among the chitin lamina when growing. Additionally, there is this question: where and how can those hyphae inside the cuticle obtain nutrition for further growth? Inside the cuticle, the concentration of reactive oxygen species (ROS) should be high due to the occurrence of melanization (**Figures 1**, **3**). Furthermore, if the penetration direction is not directly towards the hemocoel, some hyphae (e.g. hyphae in **Figures 4B, F**) may have to travel inside the cuticle for a long time. Therefore, hyphae inside the cuticle could experience serious stress that is different from that in the hemocoel. Currently, we are optimizing the conditions for transcriptome analysis of invading hyphae in order to understand their development after reaching the cuticle.

Another unexpected result is that fungal infection can induce many epidermis cells into proliferation through DNA duplication and cell division. When penetrating the integument, it is possible for some hyphae to touch the epidermis cells directly as shown in **Figure 3D**. This type of mechanical damage may promote the corresponding cell and even neighboring cells into proliferation. Apoptosis and ROS may also cause epidermis cells to proliferate. However, apoptotic cells were not detected using the Terminal deoxynucleotidyl transferase (TdT) dUTP Nick- End Labeling (TUNEL) method. NAC and vitamin C were fed to the larvae that received the fixed-site infection to decrease ROS levels, but no significant change was observed with BrdU incorporation and PH3 staining. Therefore, apoptosis and ROS are not responsible for inducing epidermis cells to proliferate. Very interestingly, the non-infection segment that was two segments away from the infection site (as shown in **Figure 1A**) could also receive signals to promote epidermis cells into proliferation (**Figures 5**, **6**). Clearly, it is a systematic response for epidermis cells to enter the cycle of DNA duplication and/or cell division. Unfortunately, there is no proper genetic method to inhibit DNA duplication and/or cell division in the silkworm epidermis cells and so it is necessary to feed larvae with Cisplatin. After inhibiting epidermis cell proliferation at a dose that does not impact silkworm growth and *B. bassiana* germination and development, the fixed-site infected larvae all quickly died at ~72 h (**Figure 7B**). Therefore, epidermis cell proliferation after fungal infection is essential for protecting the hosts from infection. Again, this phenomenon is impossible to be detected with traditional infection since it is difficult to locate the exact infection sites.

Taken together the fixed-site infection model is a convenient way to track the invading hyphae inside insect integuments that can not readily be performed using the traditional infection method. We also determined that conidia germination is dose-dependent, and melanization occurs before new hyphae develop and penetrate the cuticle. Most important of all, epidermis cell proliferation is the first important step for host defense against fungi infection. All of these phenomena are hard to detect if the traditional infection method is utilized.

## Data Availability Statement

The original contributions presented in the study are included in the article/**Supplementary Material**. further inquiries can be directed to the corresponding authors.

## Author Contributions

WH, RT, LYG, SL, YZ, RC, LG, XW, GP performed experiments. WH and EL analyzed the data. BTB and EL designed and wrote the paper. All authors contributed to the article and approved the submitted version.

## Funding

This work was supported by the National Natural Science Foundation of China, Science and Technology Commission of Shanghai Municipality, the State Key Lab of Silkworm Genome Biology under Grants (32070495, 19ZR1466500, 32070507, 31872294, 2019P02).

## Conflict of Interest

The authors declare that the research was conducted in the absence of any commercial or financial relationships that could be construed as a potential conflict of interest.

## Publisher’s Note

All claims expressed in this article are solely those of the authors and do not necessarily represent those of their affiliated organizations, or those of the publisher, the editors and the reviewers. Any product that may be evaluated in this article, or claim that may be made by its manufacturer, is not guaranteed or endorsed by the publisher.

## Supplementary Material

Supplementary Figure 1 Fixed-site infection by ΔBPS8 induced limited DNA duplication. Wild type **(A)** and ΔBPS8 mutants **(B)** were applied at the same time for fixed-site infection respectively. DNA duplication was detected at 72 h post infection with the ΔBPS8 mutant inducing weak infection. The arrows point to cells showing BrdU incorporation.

Supplementary Figure 2 Fixed-site infection did not induce apoptosis in epidermis. Larvae on day 1 of the 5th instar stage received a fixed-site infection and were sampled as described above. Cell apoptosis in epidermis was detected using the TUNEL method. No apoptotic cells were detected in the control **(A, C)** and fixed-site infection samples **(B, D)** at different time points.

Supplementary Figure 3 Cisplatin inhibits DNA duplication in a dose-dependent manner. No DNA duplication and cell division were detected in epidermis cells of larvae at the 5th larval stage (17). DNA duplication occurs in midgut cells during the 4th larval stage. Consequently, varying amounts of Cisplatin were injected into larvae on day 1 of the 4th larval stage for different periods of time. The midguts were dissected to assess DNA duplication. Compared with the control **(A)** and those with smaller amounts of Cisplatin injection **(B, C)**, 7 µg of Cisplatin clearly inhibited DNA duplication within 9 h. Some of the BrdU-positive nuclei (Red) are indicated by arrows. Nuclei were counter-stained by DAPI.

Supplementary Figure 4 Cisplatin feeding did not interrupt plasma phenoloxidase (PO) activity. Cisplatin at different concentrations was fed to larvae at the 5th larval stage for 48 h. Plasma of each larva was obtained for assaying PO activity. Cisplatin feeding did not change PO activity significantly. PO activities among different concentrations of Cisplatin feeding were almost at the same level. Data represent the average of at least 8 individuals (mean ± SE). One way ANOVA with Tukey’s multiple comparisons test was performed.
