# Supplementary figures and images for: Involvement of Epidermis Cell Proliferation in Defense Against Beauveria bassiana Infection
Source: Front Immunol. 2021 Sep 16;12:741797. doi: 10.3389/fimmu.2021.741797 (PMC8481689; doi:10.3389/fimmu.2021.741797)

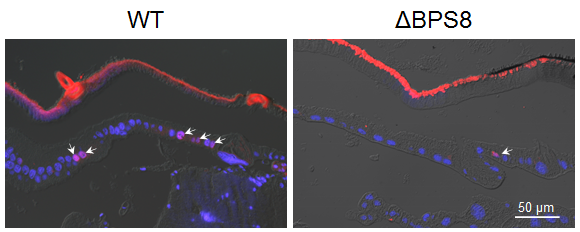

Supplement: Supplementary Figure 1 — Fixed-site infection by ΔBPS8 induced limited DNA duplication. Wild type (A) and ΔBPS8 mutants (B) were applied at the same time for fixed-site infection respectively. DNA duplication was detected at 72 h post infection with the ΔBPS8 mutant inducing weak infection. The arrows point to cells showing BrdU incorporation. [file Image_1.tif]

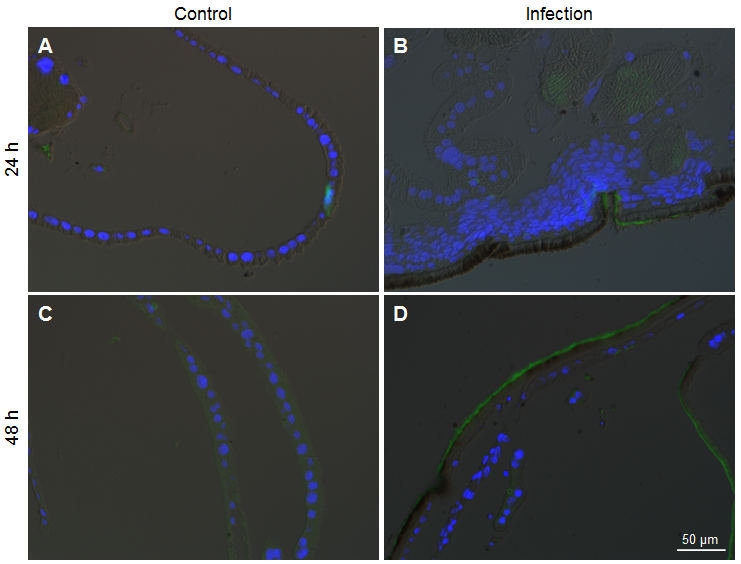

Supplement: Supplementary Figure 2 — Fixed-site infection did not induce apoptosis in epidermis. Larvae on day 1 of the 5th instar stage received a fixed-site infection and were sampled as described above. Cell apoptosis in epidermis was detected using the TUNEL method. No apoptotic cells were detected in the control (A, C) and fixed-site infection samples (B, D) at different time points. [file Image_2.tif]

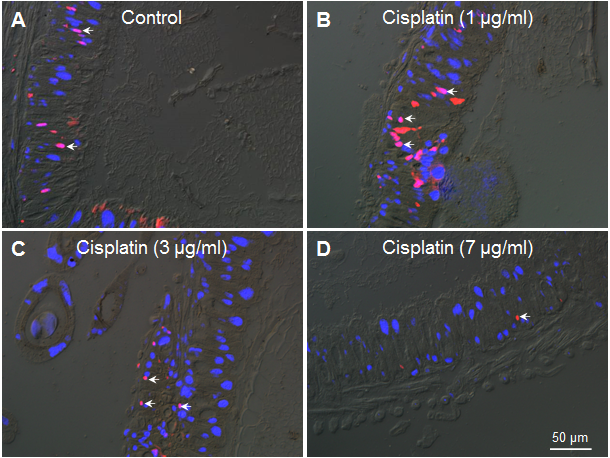

Supplement: Supplementary Figure 3 — Cisplatin inhibits DNA duplication in a dose-dependent manner. No DNA duplication and cell division were detected in epidermis cells of larvae at the 5th larval stage (17). DNA duplication occurs in midgut cells during the 4th larval stage. Consequently, varying amounts of Cisplatin were injected into larvae on day 1 of the 4th larval stage for different periods of time. The midguts were dissected to assess DNA duplication. Compared with the control (A) and those with smaller amounts of Cisplatin injection (B, C), 7 µg of Cisplatin clearly inhibited DNA duplication within 9 h. Some of the BrdU-positive nuclei (Red) are indicated by arrows. Nuclei were counter-stained by DAPI. [file Image_3.tif]

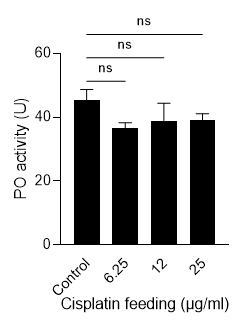

Supplement: Supplementary Figure 4 — Cisplatin feeding did not interrupt plasma phenoloxidase (PO) activity. Cisplatin at different concentrations was fed to larvae at the 5th larval stage for 48 h. Plasma of each larva was obtained for assaying PO activity. Cisplatin feeding did not change PO activity significantly. PO activities among different concentrations of Cisplatin feeding were almost at the same level. Data represent the average of at least 8 individuals (mean ± SE). One way ANOVA with Tukey’s multiple comparisons test was performed. [file Image_4.tif]
